# I-DECIDE: A Social Prescribing and Digital Intervention Protocol to Promote Sexual and Reproductive Health and Quality of Life among Young Cape Verdeans

**DOI:** 10.3390/ijerph18030850

**Published:** 2021-01-20

**Authors:** Andreia Costa, Susana Mourão, Osvaldo Santos, Violeta Alarcão, Ana Virgolino, Paulo Nogueira, Marlinda Rocha Bettencourt, Suely Reis, Albertino Graça, Adriana Henriques

**Affiliations:** 1 Instituto de Saúde Ambiental (ISAMB), Faculdade de Medicina, Universidade de Lisboa, Avenida Egas Moniz, 1649-028 Lisboa, Portugal; susanasofiamourao@gmail.com (S.M.); osantos@medicina.ulisboa.pt (O.S.); violeta_sabina_alarcao@iscte-iul.pt (V.A.); avirgolino@medicina.ulisboa.pt (A.V.); pnogueira@medicina.ulisboa.pt (P.N.); ahenriques@esel.pt (A.H.); 2 Escola Superior de Enfermagem de Lisboa (ESEL), 1600-096 Lisboa, Portugal; 3 Unbreakable Idea Research, Lda, 2550-426 Painho, Portugal; 4 Centro de Investigação e Estudos de Sociologia, ISCTE-Instituto Universitário de Lisboa (ISCTE-IUL), Avenida das Forças Armadas, 1649-026 Lisboa, Portugal; 5 Delegacia de Saúde de São Vicente, Ministério da Saúde e da Segurança Social, Mindelo 2110, Cape Verde; marlindarocha@gmail.com; 6 Universidade do Mindelo, Mindelo 2110, Cape Verde; suely.reis@uni-mindelo.edu.cv (S.R.); reitor@uni-mindelo.edu.cv (A.G.)

**Keywords:** sexual and reproductive health, social prescribing, digital intervention, community health

## Abstract

Cape Verdean governments have intensified the investment on the National Reproductive Health Program, aiming to provide universal and qualified services, especially to the youngest people. Nevertheless, data suggest that some health challenges remain in this group (e.g., high rates of early/unplanned pregnancies, illegal abortions, sexual risk behaviors). In this paper, we present a protocol of a community-based social prescribing and digital intervention to promote wellbeing and quality of life across the life course of young Cape Verdeans, with a specific focus on Sexual and Reproductive Health (SRH) related behaviors. The intervention program, to be developed in three years, will follow an Intervention Mapping approach, namely regarding needs assessment and study’s protocol. The program’s implementation and evaluation will occur simultaneously. The main expected result is the development of a sustainable training program implemented in coproduction with Cape Verdeans from Mindelo (in São Vicente island), with replicable potential in other Cape Verdean regions. The intervention will contribute to SRH-related literacy through the digital health literacy materials and to quality of life across the young’s life course.

## 1. Introduction

In the last two decades, Cape Verdean governments have intensified the investment on the National Reproductive Health Program, aiming to provide universal and qualified services to adolescents, young people, and adults, in partnership with schools and youth centers (i.e., local associations where several sporting, cultural and community activities are developed) [1]. Around 20% of the Cape Verdean population is young (15–24 years old) and specific attention has been given to Sexual and Reproductive Health (SRH) of this group (e.g., through the creation of SRH Centers for teenagers and young people, and the inclusion of sex education in the scholar curriculum) [2]. Nevertheless, some data indicate the persistence of SRH problems, especially among the youngest population, namely: (a) premature initiation of sexual life and considerable high rates of early and unplanned pregnancies; (b) adoption of risk behaviors associated with sexual life initiation, such as alcohol consumption; (c) estimates of high rates of illegal abortions, linked to physical, economic, and cultural obstacles to its implementation inside the national health service; (d) insufficient knowledge about the HIV infection despite some improvements on recent indicators about the use of condoms [1,2,3,4]. 

The prevalence of teenage pregnancies and fertility-related health problems are among the most serious social problems in Cape Verde. According to the Cape Verdean Demographic and Reproductive Health Survey II (2005), about 19% of young women aged between 15 and 19 years old got pregnant at least one time, 15.2% were already mothers, and 3.7% were pregnant. There was a poor knowledge on and little use of contraceptives; likewise, the use of a contraceptive methods in the first sexual intercourse has been reported by only 20% girls and 18% boys [5]. Taking these epidemiological scenarios into account, we propose to develop, implement, and evaluate a community-based intervention program to promote better SRH related behaviors, and, ultimately, SRH-related wellbeing and quality of life across the life course of young Cape Verdeans (i.e., 15–24 years; [6]). The implementation and effectiveness assessment will be conducted in the city of Mindelo, as a pilot for national implementation. The intervention will be tailored for relevant subgroups (with identified health risk behaviors) and will be gender- and age-sensitive.

This health promotion program will be also based on successful and already tested health interventions for improving SRH of young people (including Cape Verdean youngsters) [7], adapted to the needs and cultural-embedded values of the Cape Verdean young population, taking into account the perceptions of local health and educational professionals. A key point will be the cooperation with the community, through a social prescribing approach, i.e., an innovative intervention in primary healthcare units that allows a partnership to work with sources of support within the community. Within this perspective, the wider contextual determinants of health are important factors to address, as they that have a particular relevance on SRH behaviors [8]. Moreover, there is a growing evidence of mobile health interventions (mHealth) as cost-effective tools for improving youth SRH related behaviors, namely in low-to-middle income countries, especially because its specific advantages on minimizing youngsters’ barriers on accessing SRH services [9]. Therefore, the coproduction and use of digital health solutions will be another key component of this program, to be implemented according to the complex interventions model [10]. The idea is that the intervention could benefit from both recognized advantages of social prescribing and digital tools on promoting health behavior change and the long-term wellbeing/quality of life.

This intervention program will adapt to the Cape Verdean sociocultural context a set of good health promotion practices specifically developed in Portugal, benefiting from previous close partnership between academic and health institutions from Portugal and Cape Verde (e.g., Projeto Bairro Feliz (Happy Neighborhood’s Project)), mostly involving immigrants and young people) [11]. A main assumption of this project regards to the potential of health and educational professionals on minimizing barriers to access to health services and on increasing communities’ health literacy and their capacity to avoid risky behaviors, through individual’s education and fostering health-oriented social activities. The behavioral change wheel will support the intervention development [12,13].

Considering that SRH is key to improve the quality of life across the life course [14], the proposed intervention will focus on promoting lifelong SRH-related wellbeing and quality of life. Due to the several definitions of quality of life and wellbeing, we will embrace a comprehensive approach, which includes not only the physical, mental, and social health but also life satisfaction (overall quality of life) [15]. Similarly, the SRH-related wellbeing is here conceptualized and will be assessed according to a socioecological approach, i.e., as intraindividually experienced SRH-related wellbeing but also socially and structurally influenced [16]. Thus, we intend to improve the youth’s quality of life and wellbeing through this community-based intervention program, considering short-term outcomes at a multidimensional perspective:

**Hypothesis** **H1 (H1).** At the individual level and self-care domain—increase in youth’s perceived general self-efficacy, assertiveness, sense of coherence, and self-esteem related to SRH behaviors and decision taking;

**Hypothesis** **H2 (H2).** Related to mental health and wellbeing domain—improvement in stress coping/resilience skills related to SRH;

**Hypothesis** **H3 (H3).** At interpersonal and social domain—higher perceived social support (and related satisfaction) and improvements in the social cohesion relate to SRH.

**Hypothesis** **H4 (H4).** In the subjective life domain—higher levels of happiness and satisfaction with life throughout adolescence and adulthood;

Several of these short-term expected outcomes (e.g., sense of coherence, self-esteem, perceived stress) were also assessed in other salutogenic health promotion programs [17], due to be considered as relevant determinants of health behavior change. Figure S1 details its specific role in the context of the proposed intervention, by schematically represent and detail the I-Decide conceptual rational. 

According to the theory behind the proposed intervention, improvements on youngsters’ adherence to healthy sexual and reproductive behaviors, cognitive function, health literacy (intermediate-term), and also in their physical health and psychosocial well-being (long-term) are also expected.

## 2. Materials and Methods

Considering the complex challenge of health behaviors change interventions, namely, on the SRH area, an Intervention Mapping plan will systematically guide the program’s development and coordination [18,19]. The steps of this Intervention Mapping protocol will correspond to the proposed four main components of our program (Table S1), as identified and described below. 

Overall, the intervention will be developed within a timeline of three years, in which needs assessment and program’s protocol development (Components 1 and 2) will be implemented subsequently. Afterwards, the program’s implementation and evaluation (Components 3 and 4) will occur simultaneously, as an evidence-based local pilot intervention that could be afterwards extended to a national implementation. 

### 2.1. Component 1—Needs Assessment of SRH among Cape Verdean Youngsters

The aim of this component is to identify the most relevant SRH-related needs, specific to youngsters’ groups and target behaviors to address in the Cape Verdean context, through focus groups (FG) sessions with main stakeholders (i.e., Cape Verdean youngsters and local health and educational providers) [10]. It starts at the beginning of the program and is expected to develop for 9 months.

To support the sociocultural adaptation of good practices tested in Portugal [11], two FG will be conducted in the country: one FG with 15–24 years old Cape Verdeans immigrants who already benefited from SRH interventions and the other with health professionals involved in those interventions. We also foresee a minimum (aiming to achieve data saturation) of 4 FG with youngsters and 4 FG with educational and health professionals (e.g., GPs, nurses, psychologists, high school teachers), in Cape Verde. Each FG will include 8 participants [20]. Sampling will follow intentional theoretical criteria: socioeconomic strata, educational levels, gender, age groups. Recruitment will be conducted by snowball methods while ensuring participants’ heterogeneity. 

Data from FG (audio-recorded and verbatim transcribed) will be analyzed through descriptive thematic analysis [21], assisted by MAXQDA, and visually depicted in a Logic Model, which schematically represents the individual and environmental determinants of Cape Verdean SRH risk behaviors in relation to the health problem(s) intended to be reduced/eliminated (e.g., strong cultural/societal values around family and childbearing that contribute to early or unintended pregnancy) [18]. In addition, based on the results of the needs assessment, the proposed program’s outcomes (e.g., perceived social support, self-efficacy, stress coping/resilience, assertiveness, happiness, satisfaction with life) will be confirmed and better specified, in order to assess the effectiveness of the intervention (Component 4).

### 2.2. Component 2—Delphi Procedure for Designing the Intervention Protocol and Components

A Delphi expert-consensus technique will be used, as an approach to enhance effective decision-making in health care. The Delphi methods has some advantages regarding other methods for obtaining expert opinion and consensus (e.g., expert round tables) [22,23]. This component is expected to last 9 months and aims to map actions responding to SRH-related needs, detected in Component 1 [10]. 

The Delphi panel will include social workers, public health professionals (e.g., nurses, general practitioners, psychologists), academics, members from national and international nongovernmental organizations (NGOs), and policy makers (e.g., working on the national health and youth ministries). Panel experts should have, at least, five years of professional experience on the youth SRH area in Cape Verde. The sample size for constructing a Delphi panel will be oriented by the effort to maximize expertise about the Delphi subject of interest (homogeneity criteria, also ensuring to involve all relevant experts in the specific area), together with different and complementary perspectives (heterogeneity criteria). Accordingly, the recruitment will be conducted by using snowball methods, being expected that the professionals recognized by their peers as experts on SRH will be identified and then invited to participate in the project. Due to the snowball sampling procedure, it is not possible to indicate a sample size that may be considered inclusive enough. Ultimately, the sample size will be defined by the panel itself. Anyway, a sample size of at least 25 experts will be required for guaranteeing a stability of answers (maximum of 50 items), as proposed by Ralitsa et al. [24].

The Delphi method will follow a minimum of two rounds. The panelists will fill in a questionnaire about good practices in SRH. Data collection form will include two types of questions:

1. Related to participants’ agreement about different paradigms, models, contents and skills to promote among (youth) SRH, using a 5-point Likert scale (1 = totally disagree to 5 = totally agree);
2. Regarding operational aspects of the intervention (e.g., number, frequency, duration and locals of activities), using single choice answer formats.

Throughout the questionnaire, the experts will be also encouraged to make comments, propose new items, and/or reformulate the items they attributed a low score to (i.e., “1-, 2 or 3” on a 5-point scale; “Not relevant” in a binary response).

At the final of each round, the item’s consensus level will be calculated in order to decide about its approval (with predefined consensus criteria for each indicator). Once again, questions about participants’ sociodemographic and professional-related information will be collected and considered for the analysis.

This component will result in the design of the intervention program’s protocol, with the identification of the theory-based behavior change methods that better apply to the previously identified SRH problems of our target group and the decision on how to better apply them [10,18,25]. Thus, the draft of the program will emerge theoretically, but specially, from this Delphi study will be fine-tuned a-posteriori by local community partners and by the target group, minimizing resistance to its implementation, according to the proposed community-based participatory approach [26].

### 2.3. Component 3—Program’s Implementation: Social Prescribing and Digital Health Activities

The implementation of the intervention will be informed by the work developed within the previous components and is expected to last 12 months. The baseline framework of the intervention is supported by a combination of Social Prescribing and digital health literacy activities [8,9,10]. As a starting point, primary health care professionals (e.g., nurses, general practitioners, psychologists) will assess young patients during their interventions/consultations and will refer them to a facilitator/navigator (e.g., a Cape Verdean doctoral student) that will then provide individual support to access nonmedical/community resources of SRH-related health behaviors (e.g., educational professionals, local associations) [27]. 

A minimum of 2500 youngsters and of 100 professionals will be invited to participate in at least one of the activities of the program. With this procedure is assumed that it will be possible to retain at least 400 youngsters in the implementation, which will allow for the detection of relatively small differences of the intervention.

The intervention will consist in the involvement of the young Cape Verdeans in prescribed social and digital health related activities, which will be mainly defined in cocollaboration both with the target group and the community partners, due to the proposed community-based participatory approach [26]. Considering other developed and tested promotion programs for young people in SRH area, it may include activities such as the involvement in creative art groups and in a final Portuguese-Cape Verdean exhibition about health SRH-related competences, enhancing the quality of life across the life course and positive learning about SRH salutogenic experiences. In the involved community institutions, they can collaborate in the coproduction of some digital information about SRH, to be included in a SMS and web-based question-and-answer service, promoting content and critical SRH literacy. In addition, in the coproduction of m-health promotion tools that could be afterward used by themselves or by other young Cape Verdean peer (e.g., a free mobile quiz game about SRH contents in which participants could win credits to make calls) and that facilitate knowledge sharing and SRH behavior change [9,28].

Activities related with the program’s dissemination are also planned, aiming to facilitate networking and communication among the scientific community, health professionals, and the general public [29]. These outreach initiatives will start three months after the beginning of the project because it will imply the production of materials, such as: a logo and an intervention’s brochure (visit card of the program); a program’s website with informative materials with an interactive component (social media profiles on Facebook, LinkedIn, Instagram, Twitter and ResearchGate).

### 2.4. Component 4—Program’s Evaluation with RE-AIM Framework

The program’s evaluation will be based on the RE-AIM framework [30], which will measure the impact of this community-based intervention in five different dimensions:

1. Reach—assessment of the percentage of youths contacting with the program (getting aware with it, getting involved in any way with it);
2. Effectiveness—assessed with the noncontrolled community trial;
3. Adoption—will evaluate some process-related outcomes, detailed below;
4. Implementation—will evaluate the extent to which the intervention is adequate and is accomplished across the intervention (fidelity);
5. Maintenance of qualities/properties—will evaluate the extent to which the intervention will be sustained over time.

This task will occur simultaneously to the program’s implementation (i.e., during 12 months), but with three specific key moments: at baseline time and at two different follow-up moments (3 months and 6 months after being involved) [7]. Considering the Social Prescribing component of the program, participants will include youngsters that were previously referred to the nonmedical sources of SRH-related health behaviors (e.g., local associations) and so they will be recruited in these community contexts [27]. Expecting that only 1 out of 5 individuals will accept to participate, the baseline sample size will be composed of about 500 youngsters. Considering additionally a 20% potential dropout between baseline and the second follow-up, we estimate a minimum final sample size of 400 youngsters.

According to the previous contextualization, our proposal is to develop an intervention focused on the improvement of the long-term young Cape Verdeans’ SRH-related wellbeing and quality of life, by decreasing their SRH problems and barriers to SRH behavior change, also by increasing their SRH related literacy. This medium- and long-term impact will be assessed in a short-term by the outcomes detailed above (i.e., H1 to H4). The proposed short-term outcomes will be assessed by specific translated and/or cross-culturally validated scales. To assess outcomes at the individual level and self-care domain (H1), the following measures will be used:

1. Portuguese version of the Self-Efficacy Scale, a 15-item measure that evaluates the general self-efficacy, throughout three specific subdimensions—initiation and persistence (6 items; e.g., *I’m a self-confident person*), efficacy to face the adversity (5 items; e.g., *I easily give up on things*) and social efficacy (4 items; e.g., *My friendships have been achieved through my personal abilities to make new friends*). Participants’ answers are rated on a 7-points Likert Scale (1-totally disagree to 7-totally agree) [31].
2. Portuguese version of the Antonovsky’s Sense of Coherence Scale (SOC 13) [32], a measure that assesses the main construct of the salutogenic theory, the sense of coherence, through three high interrelated subdimensions–comprehensibility (5 items; e.g., *Do you usually feel that the things that happen to you in your daily life are hard to understand?*), manageability (4 items; e.g., *Do you usually see a solution to problems and difficulties that other people find hopeless?*) and meaningfulness (4 items; e.g., *Do you usually feel that your daily life is a source of personal satisfaction?*). It also uses a 7-point scale that is anchored by two phrases [33].
3. Portuguese version of the Rosenberg Self-Esteem Scale (RSES), a 10-items unidimensional measure that evaluates the adolescent and adults’ self-esteem. Examples of typical items are: “*On the whole, I’m satisfied with myself*” or “*I feel I’m a person of worth*”. Participants’ answers are rated on a 4-points Likert Scale (0-strongly disagree to 4-strongly agree) [34].

To assess outcomes at social and interpersonal level (H2), three different instruments will be used, namely:

1. Portuguese version of the Multidimensional Scale of Perceived Social Support, a 12-items measure that assesses the subjective social support at three main areas—family (4 items; e.g., *My family truly tries to help me*), friends (4 items; e.g., *I can count on my friends when something goes wrong*), and other significant (4 items; e.g., *There is a special person with whom I can share my happiness and sadness*) [35]. Participants’ answers are rated on a 7-points Likert scale (1-totally disagree to 7-totally agree).
2. Portuguese brief version of Satisfaction with Social Support Scale, also a 12-items measure composed by two specific subdimensions—satisfaction with social support (7 items; e.g., *I’m satisfied with the number of friends that I have*) and need for activities related to social support (5 items; e.g., *I miss social activities that satisfy me*). The young Cape Verdeans must indicate the degree to which they agree with each affirmation (if it applies to them) on a Likert scale with five answer options [36].
3. A Social-Cohesion Index (VALCOS), a macro index of social cohesion that covers indicators of several dimensions of social life, including health and subjective well-being [37].

To measure outcomes related to mental health care (H3) we will use the Portuguese version of the Brief Resilient Coping Scale, composed by four items (e.g., *I look for creative ways to overcome difficult situations; I actively look for ways to replace losses that I have in life*) and where the participants’ answers are assessed by a 5-points Likert-type scale (1-Almost never to 5-Often) [38]. 

Lastly, subjective wellbeing domain (H4) data will be assessed through the Portuguese version of the Lyubomirsky and Lepper Subjective Happiness Scale. This is a 4-times measure, with a 7-points visual scale anchored in two antagonistic statements, which express the participants’ level of happiness (e.g., *Generally, I consider myself as a: 1-not very happy person to 5-very happy person*) [39].

The program’s adoption will be assessed by the percentage of health professionals conducting social prescribing and the number of youngsters getting a social prescribing. Similar to other Social Prescribing interventions, implementation and reach assessment will be measured by the youth’s level of engagement with the intervention and by their satisfaction with the program [40]. Finally, the extent to which the intervention is sustainable—maintenance—will be evaluated by the willingness from involved health and educational professionals, as well as from community resources stakeholders, in training peers from other regions.

As presented in Figure S1, several contextual, social, and cultural conditions from young Cape Verdeans may influence their SRH behaviors (i.e., gender, ethnicity, and socioeconomic conditions, such as education and household income). Thus, they will be controlled in all the sampling and analysis procedures. 

### 2.5. Statistical Analysis

The main interest in this study relies on comparing individuals’ characteristics between baseline and follow up moment subsequent to intervention. Considering generic continuous characteristics in classic parametric context, a sample size of *n* = 393 will allow detecting a minimum standardized difference of 0.2, with 80% statistical power and a 95% confidence level.

To assess statistical significance of intervention, the paired-sample T test or the nonparametric Wilcoxon signed-rank test will be used. Additionally, multivariable analyses will be performed using general linear models (GLM) with normal distributed errors to study the factors and covariates that associate with the differences of individuals characteristics before and after intervention.

### 2.6. Ethical Considerations

The intervention will be conducted in accordance with the Declaration of Helsinki, and the presented protocol was approved by the Cape Verdean National Commission of Data Protection (process number 296/2020/CNPD). After its approval, the protocol was also submitted to the Cape Verdean Health Research Ethics Committee, as it was defined by the country. 

Participants will give their written consent to participate after being fully informed about each component’s objectives and activities, anonymity/confidentiality of the data collected, and the voluntary nature of their participation. In the case of participants who are 15 to 17 years old, the written consent from parents or a legal representative will be also asked.

## 3. Expected Results and Outputs

As previously detailed, the main results of the proposed program are the long-term promotion of young Cape Verdeans SRH-related wellbeing and quality of life. As intermediate results, and constituting the short-term indicators of the major results, a positive impact is also expected in crosscutting youth competencies from different dimensions such as: individual level and self-care domain (i.e., perceived general self-efficacy, sense of coherence, and self-esteem); social and interpersonal level (i.e., perceived social support and related satisfaction), social cohesion and assertiveness); mental health (i.e., stress/coping resilience skills); subjective wellbeing domain (i.e., happiness and satisfaction with life). 

The program’s implementation will also result in different forms of data integration and presentation. It is expected that the intermediate results from each component will contribute to the production of technical reports and scientific papers. The preliminary findings from these papers will be also communicated and disseminated through the team’s participation in national and international conferences in the fields of SRH and public health. Another important output will be the production of advanced (sustainability-oriented) health professionals training materials, namely a standard operational procedure manual for the reimplementation of the intervention program.

In addition to the production of training materials and standard operating procedures for the intervention implementation, the sustainability of this intervention program will be also enhanced thanks to the potential for replication of the study with other populations and wide use of the materials produced. 

Mainly resulting from the program’s implementation (Component 3), a biennial Portuguese-Cape Verdean exhibition about SRH-related literacy, co-organized by health professionals, educational professionals, and youngsters, will also constitute a relevant intervention’s output. Similarly, the joint production of digital health literacy materials is expected, including at least one free mobile quiz game about SRH and quality of life across the life course. This program output could be used afterwards by intervention users or adapted to their peers (including those from other Cape Verdean regions or other African countries).

## 4. Discussion

The proposed program can have a meaningful impact on the consolidation of the intervention and research capacity in Cape Verde. It has a plan of action for the establishment of networks of researchers, health and educational professionals, and research cooperation between Portugal and Cape Verde, namely through the translation to the Cape Verdean sociocultural context of good health promotion practices specifically developed and tested in Portugal. Accordingly, this involvement will target improvements in research and intervention competencies for at least a Cape Verdean promising researcher, which may guarantee the portability (to other cities/regions of Cape Verde) and sustainability of the program over time. The extent to which the intervention will be sustained over time will be also evaluated by measuring the willingness of the involved health professionals/head of community resources in training their peers to replicate the intervention in other Cape Verdean regions; especially because its implementation and assessment will be conducted in the city of Mindelo but has a pilot for national implementation. A standard procedure manual, developed as advanced and sustainability-oriented health and educational professionals training materials, will not only allow the adequate evaluation of the intervention effectiveness but also the adequate reimplementation of the good practices that will prove as effective in Cape Verde sociocultural context throughout this project.

Potential future achievements are also planned, demonstrating the added value of the proposed cooperation, namely through some of the social prescribing and digital health literacy activities that will be implemented throughout the program. Accordingly, one of the expected outputs is that a biennial exhibition about SRH-related literacy could be co-organized by health professionals, educational professionals, and youngsters both from Portugal and Cape Verde. Besides this, it is expected that a quiz mobile game about SRH and quality of life across the life course, coproduced with the Cape Verdean young participants in the first phase of the intervention, could be afterward used by themselves or adapted to their peer (including those from other African countries or regions).

Globally, the proposed intervention is focused on having a meaningful impact on the quality of life in Cape Verde, namely considering that the young Cape Verdeans universal access to SRH is key to improve their quality of life across a life course perspective [5]. This SRH promotion targets the development of social cohesion perception, throughout the articulation and strait cooperation between health and educational resources with other public or private agents (e.g., arts-related organization), within the framework of social prescribing. The visibility of such cohesion between stakeholders is expected to have a positive impact also in social support perception [41]. Both social prescribing and digital health related initiatives will target the promotion of crosscutting youth competences (e.g., health-related locus of control, stress coping/resilience, assertiveness, self-esteem) that has positive impact in a wide variety of human life course activities, beyond SRH, that, ultimately, may contribute to highly relevant gains of quality of life at individual and community levels.

Summing up, we will embrace a comprehensive approach of well-being and quality of life, bearing in mind not only the young Cape Verdeans’ individual, mental, and social health but also their life satisfaction and some of their contextual elements (overall quality of life).

Some limitations of this study are related with the specific context of one island (small, social, economic, culturally specific characteristics) where the study will be developed. Another limitation concerns the risk of dropout. However, in future research the extension to other islands is possible in order to replicate the study in different contexts and with a more powerful sample to confirm outcomes. Nevertheless, the replication study from this intervention has a high potential outside of Cabo Verde related with the language context in other African country’s (e.g., São Tomé e Principe, Angola) and Brazil, with gains of quality of life at individual and community levels.

## 5. Conclusions

This study presents an added value as a health promotion intervention with a comprehensive approach of well-being and quality of life across the life course.

The framework of social prescribing and digital health literacy that supports this SRH intervention will allow social cohesion, throughout the cooperation between health and educational, from public or private sectors, including relevant stakeholders. A positive impact in social support perception is also expected, which is relevant from the perspective of sustainability. It is projected that the implementation and effectiveness assessment will embrace a pilot for national implementation, allowing to benefit several Cape Verdean’s local communities, health services, educational programs, and policymakers.

## Data Availability

Non-applicable, due to be an intervention protocol, without data at this moment. Is expected that more relevant results will be disseminated through scientific papers.

## Supplementary Materials

The following are available online at https://www.mdpi.com/1660-4601/18/3/850/s1, Figure S1. I-Decide Conceptual Map; Table S1. I-Decide Intervention Mapping Plan.
