# Supplementary material for: I-DECIDE: A Social Prescribing and Digital Intervention Protocol to Promote Sexual and Reproductive Health and Quality of Life among Young Cape Verdeans
Source: Int J Environ Res Public Health. 2021 Jan 20;18(3):850. doi: 10.3390/ijerph18030850 (PMC7908165; doi:10.3390/ijerph18030850)
Supplement: Supplementary file 1 [file ijerph-18-00850-s001.pdf]

Figure S1. I-Decide Conceptual Map

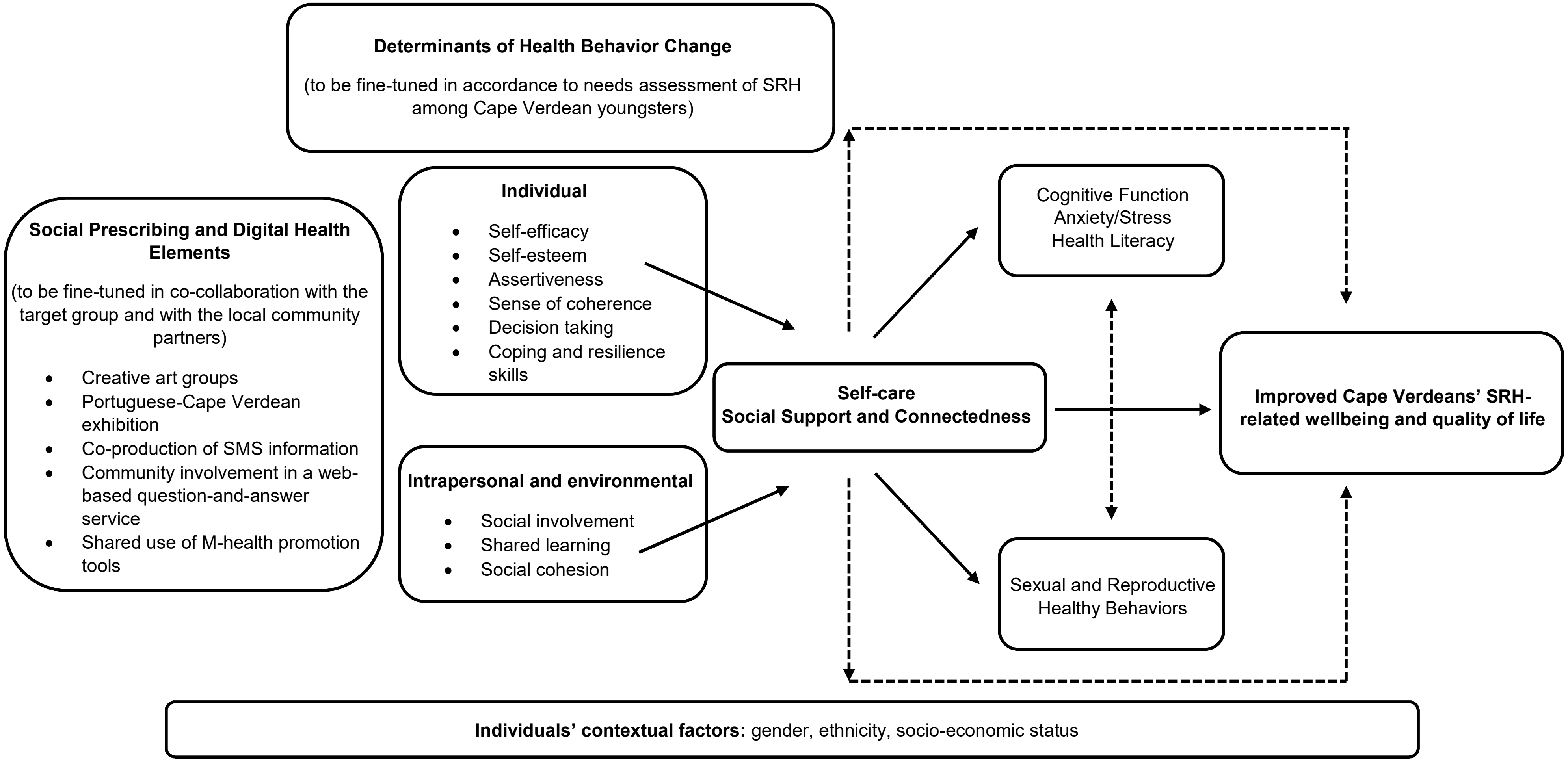

Adapted from:
Leavell, M.A., Leiferman, J.A., Gascon, M. et al. (2019). Nature-Based Social Prescribing in Urban Settings to Improve Social Connectedness and Mental Well-being: a Review. Current Environmental Health Reports, 6, 297–308. https://doi.org/10.1007/s40572-019-00251-7
McCarthy, O.L., Wazwaz, O., Osorio Calderon, V. et al. (2018). Development of an intervention delivered by mobile phone aimed at decreasing unintended pregnancy among young people in three lower middle income countries. BMC Public Health 18, 576. https://doi.org/10.1186/s12889-018-5477-7

Table S1. I-Decide Intervention Mapping Plan

Adapted from W.K. Kellog Foundation. (2004). Logic Model Development Guide

Theoretical assumptions	Inputs	Activities	Outputs	Outcomes	Impact
The prevalence of teenage pregnancies and fertility-related health problems are among the most serious social problems in Cape Verde and so Cape Verdean governments have intensified the investment on the National Reproductive Health Program, in close partnership with local community institutions	SP triple hex: youngster Cape Verdean primary healthcare users, health professionals as social prescribers and community institutions as social providers	Component 1: Assessing most relevant SRH-related needs, while promoting the stakeholders' engagement with the program	Logic Model, which schematically represents the individual and environmental determinants of Cape Verdean SRH risk behaviors in relation with the health problem(s) intended to reduce/eliminate	Perceived gains for community agents to be involved in the SP network	Comprehensive and integrated primary healthcare delivery system
			Better specification of program's outcomes, in order to assess the effectiveness of the intervention		
			Materials and activities to publicly spread the project		
SP interventions are appropriate for reaching a range of high-risk populations, due to a partnership cooperation between health and community resources, in order to address and intervene in wide contextual determinants of health	Research team in partnership with local trained staff, both with expertise and leadership skills to implement the project at a local level	Component 2: Delphi procedure to design a pilot SP and digital health intervention on SRH area	Intervention protocol and respective components	Improvements in the primary health care professionals capacity to intervene on SP and SRH areas	Increased healthcare system sustainability
By referring youngsters Cape Verdean healthcare users' to local community institutions, where will be involved in the co-production/sharing of digital information/tools, healthcare professionals may contribute to positive impacts on individuals' holistic health and wellbeing, and also to better health services' sustainability		Components 3 and 4: Implementing and evaluating an evidence-based SP and digital health intervention to to promote better SRH related literacy and behaviors	Best practices on SP and digital interventions on SRH	Improvements in the youngsters Cape Verdean's primary healthcare users' indicators:<br>- Self-efficacy, self-esteem, assertiveness, sense of coherence, decision taking, coping/resilience skills, happiness, satisfaction, social support, social cohesion (short-term);<br>- Adherence to healthy sexual and reproductive behaviors, cognitive function, health literacy (intermediate-term);<br>- Physical health and psychosocial well-being (long-term)	Improved SRH-related wellbeing and quality of life across the life course of young Cape Verdeans
			Integrated results about the intervention's implementation and evaluation in the selected pilot region		
			High level of engagement and satisfaction with the SP system		
**Beginnings**	**Planned Work**		**Intended Results**		
